# Applying Faculty Practice Metrics to Residency Clinics: Implications for Continuity of Care, Patient Experience, and Resident Wellness

**DOI:** 10.7759/cureus.103086

**Published:** 2026-02-06

**Authors:** Humza F Siddiqi

**Affiliations:** 1 Family and Community Medicine, University of Texas Southwestern Medical Center, Dallas, USA

**Keywords:** continuity care, health system, health system reform, patients satisfaction, press ganey scores, resident clinic

## Abstract

Continuity of care and patient experience metrics are increasingly used by health systems to evaluate outpatient clinical performance. However, these benchmarks are often derived from faculty-only practices and applied uniformly to residency teaching clinics without sufficient consideration of structural differences inherent to graduate medical education. Residency clinics face unique challenges related to variable trainee schedules, competing educational demands, and team-based care models, all of which can negatively influence continuity measures and patient satisfaction scores such as Press Ganey.

This editorial examines the limitations of applying faculty-practice performance metrics to residency settings and highlights how misaligned expectations may inadvertently affect patient care, resident experience, and program evaluation. Drawing on current family medicine literature and operational leadership experience, the article proposes practical, systems-level strategies for contextualizing performance data, refining scheduling models, and aligning evaluation frameworks with the dual missions of education and patient care. A more nuanced interpretation of continuity and satisfaction metrics may allow health system leaders to better support residency clinics while preserving accountability and quality improvement goals.

## Editorial

Introduction

Continuity of care is a foundational principle of family medicine and a core educational objective of residency training programs. Recent literature has emphasized both the importance of continuity in residency clinics and the role of scheduling interventions in improving longitudinal patient-resident relationships [1,2]. More broadly, discontinuity in care and education has been increasingly recognized as a systemic challenge across medical training environments, with implications for patient outcomes and learner development [3]. At the same time, health systems increasingly rely on standardized continuity and patient satisfaction metrics to evaluate clinic performance. Residency teaching clinics, however, operate within structural constraints that differ substantially from faculty-only practices. When these differences are not adequately considered, performance metrics may unintentionally misrepresent care delivery in training environments.

Structural barriers to continuity in residency clinics

Residency clinics function within inherently variable schedules shaped by block rotations, inpatient responsibilities, electives, and duty-hour requirements. These necessary educational structures fragment resident availability and limit the degree of continuity achievable compared to faculty-only practices with stable clinician presence. While enhanced scheduling models can meaningfully improve continuity, they cannot fully eliminate these disruptions, particularly in clinics serving medically underserved or high-acuity populations [1-3]. Expecting parity with faculty-only continuity benchmarks may therefore be unrealistic in many training settings.

Patient satisfaction metrics and unintended consequences

Patient satisfaction tools, including widely used survey instruments, such as Press Ganey scores [4], often emphasize access, availability, and continuity. In residency clinics, patients may experience frustration when appointments are rescheduled or when their primary resident physician is unavailable due to educational obligations. These experiences can negatively influence satisfaction scores despite high-quality, team-based care. When residency clinics are directly compared with faculty-only practices, these structural differences are rarely contextualized, potentially disadvantaging teaching clinics in performance evaluations [1,3].

These dynamics are reflected in our institute's patient experience data within the Department of Family Medicine. During the 2025 calendar year, faculty-only practices achieved Press Ganey satisfaction scores exceeding 94% across more than 700 completed surveys, while resident-only clinics demonstrated lower overall satisfaction scores at approximately 75% based on over 200 surveys. In mixed teaching clinics where residents and faculty practice together, combined satisfaction scores fell between these extremes at approximately 81%. While these data are descriptive and not intended as a formal comparative analysis, they illustrate how structural features of residency training environments may influence patient experience metrics when evaluated alongside faculty-only practices.

Impact on residents

The consequences of misaligned metrics extend beyond clinic performance reports. Residents frequently experience moral distress when they are unable to provide the continuity they value and were trained to prioritize. Repeated exposure to negative patient feedback related to access or continuity factors largely outside residents’ control may contribute to professional dissatisfaction and diminished fulfillment [1,3]. Over time, this tension risks undermining both the educational environment and the sustainability of training programs.

Practice management and leadership strategies

Addressing these challenges requires a systems-based approach that extends beyond scheduling optimization alone. Effective strategies include transparent communication with patients about the structure and mission of residency clinics, intentional team-based continuity models, and thoughtful interpretation of patient experience data [1-3]. Health system and operational leaders play a critical role in ensuring that continuity and satisfaction metrics are evaluated within the context of residency training rather than applied uniformly across faculty and teaching practices. Aligning evaluation frameworks with educational missions can support patient-centered care while preserving accountability and quality improvement goals.

Conclusion

Residency teaching clinics serve a dual mission of delivering high-quality patient care while training future family physicians. Recognizing the structural realities inherent to this mission is essential when applying continuity and patient satisfaction metrics. By adopting context-sensitive evaluation strategies and leadership-informed practice management approaches, health systems can better support patients, residents, and educators alike while continuing to advance continuity as a core value of family medicine [1-3].
